# Bladder Eversion Through a Vesicovaginal Fistula in a Patient With Complete Uterine Prolapse

**DOI:** 10.1002/iju5.70064

**Published:** 2025-07-28

**Authors:** Ryoken Tsunekawa, Naoki Wada, Haruka Takagi, Tsubasa Hatakeyama, Masaya Nagabuchi, Shun Morishita, Hidetoshi Ichikawa

**Affiliations:** ^1^ Department of Renal and Urologic Surgery Asahikawa Medical University Asahikawa Japan; ^2^ Department of Obstetrics and Gynaecology Asahikawa Medical University Asahikawa Japan

**Keywords:** bladder eversion, uterine prolapse, vesicovaginal fistula

## Abstract

**Introduction:**

We report a case of bladder eversion through a vesicovaginal fistula (VVF) in an elderly patient with severe pelvic organ prolapse (POP).

**Case Presentation:**

A 90‐year‐old woman presented with a sensation of prolapse and urinary leakage. She was diagnosed with complete uterine prolapse and bladder mucosal ectropion through a VVF, with renal dysfunction due to bilateral hydronephrosis. A one‐stage minimally invasive surgical repair was performed. The VVF was then closed in two layers and reinforced with a Martius flap. Colpocleisis was performed without the addition of transvaginal hysterectomy. At 18 months after surgery, the patient remained free of POP and urinary incontinence.

**Conclusion:**

In elderly patients, VVF can develop in advanced POP. Minimally invasive treatment is desirable, and early intervention for POP may help prevent this complication.

**Abbreviations**
PCA: percutaneous coronary angioplasty
PCI: percutaneous coronary intervention
POP: pelvic organ prolapse
TAVI: transcatheter aortic valve implantation
VVF: vesicovaginal fistula

**Summary**

- Pelvic organ prolapse (POP) is often latent in very elderly women but can occasionally present as severe POP with complications such as vesicovaginal fistula and bladder eversion.
- Accurate assessment of the anatomical status is essential, and minimally invasive repair should be considered based on the patient's overall condition.

## 1 Introduction

Pelvic organ prolapse (POP), including uterine prolapse and cystocele, is a common condition in middle‐aged and elderly women. Among Japanese women aged 70 years or older, 28.7% had POP‐Q stage II or higher [1]. Vesicovaginal fistula (VVF) most commonly occurs due to iatrogenic injury during surgery or obstructed labor [2, 3]. Here, we report a case of complete uterine prolapse with bladder eversion through a VVF of unknown etiology in a very elderly patient.

## 2 Case Report

A 90‐year‐old woman (gravida 2, para 2, body mass index: 23.1 kg/m^2^) had experienced a sensation of POP for approximately 2 years but had not sought medical attention. She had a medical history of transcatheter aortic valve implantation for aortic stenosis, percutaneous coronary intervention (PCI) for angina pectoris, hypertension, and paroxysmal atrial fibrillation, for which she was under regular medical follow‐up.

**FIGURE 1:**
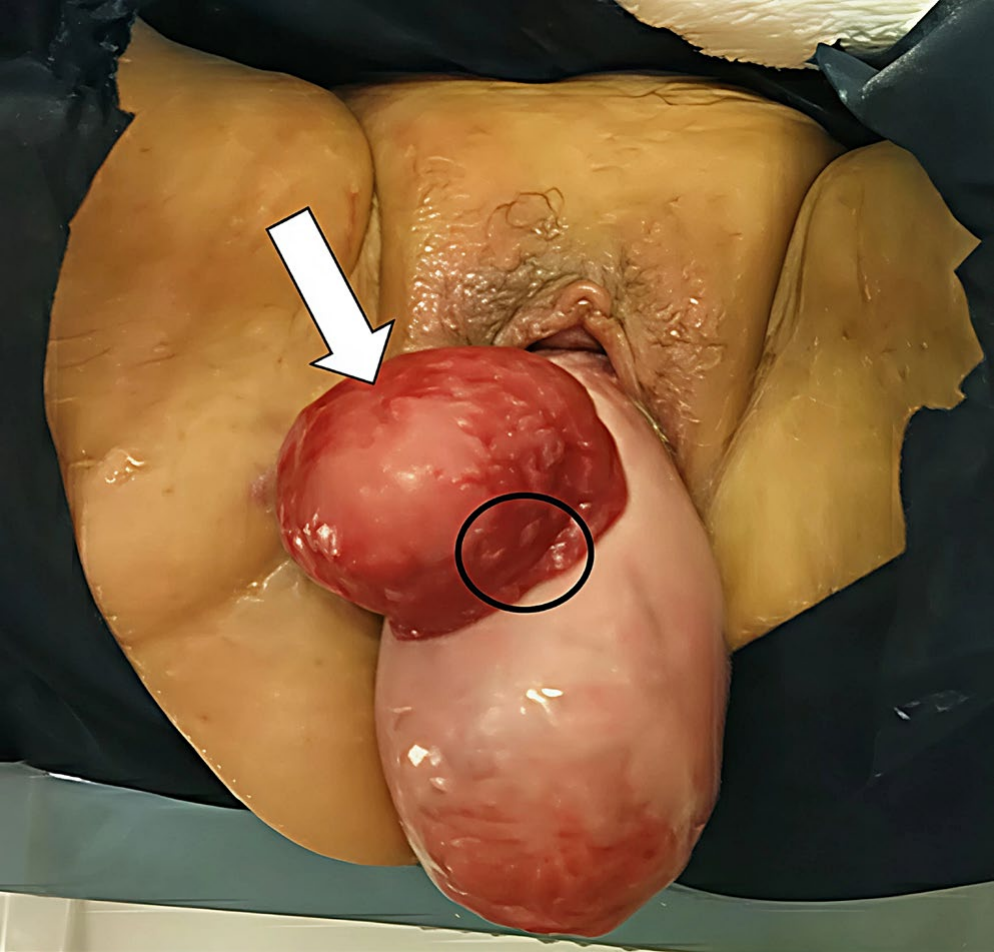
Outpatient visual examination. Complete uterine prolapse and bladder mucosal ectropion (white arrow) through vesicovaginal fistula are observed. Urine was discharged directly from the ureteral orifices (around the black circle: Left orifice). Bladder mucosa could be reparable manually, but uterine prolapse was difficult to repair due to pain.

**FIGURE 2:**
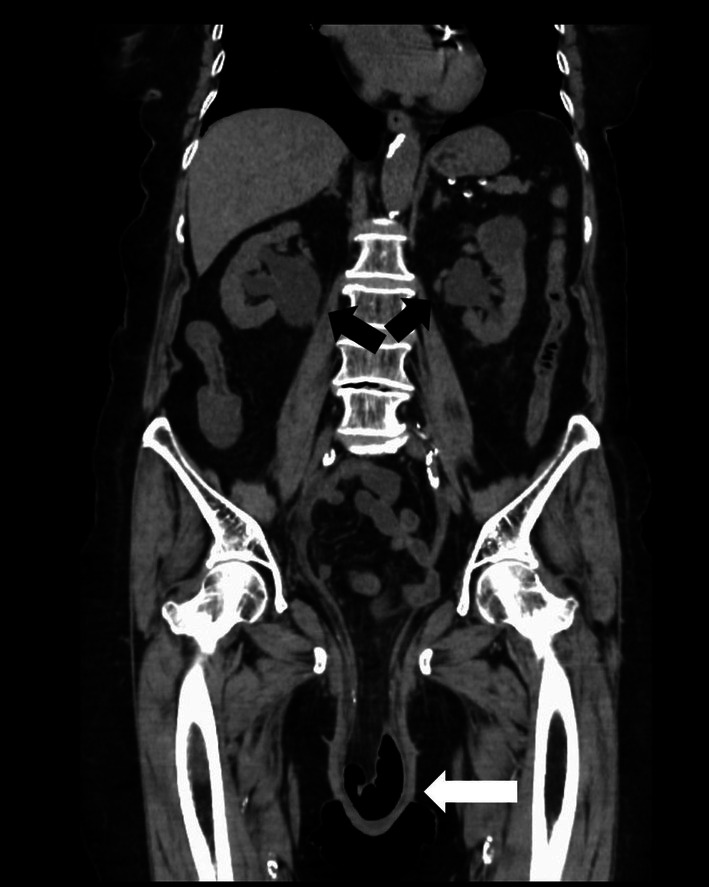
CT findings. Abdominal CT showed severe pelvic organ prolapse (white arrow) and bilateral hydronephrosis (black arrows).

The patient initially consulted a gynecologist due to a sensation of prolapse and urinary leakage. The gynecologist diagnosed uterine prolapse but referred her to our department for further evaluation of an abnormal urinary tract. A visual examination (Figure 1) revealed complete uterine prolapse and bladder mucosal ectropion through a VVF. A computed tomography scan confirmed POP and bilateral hydronephrosis (Figure 2), with a serum creatinine level of 1.81 mg/dL. Urine output was visible from the ureteral orifices. While the bladder mucosa was deemed reparable manually, repair of the uterine prolapse was difficult due to pain.

**FIGURE 3:**
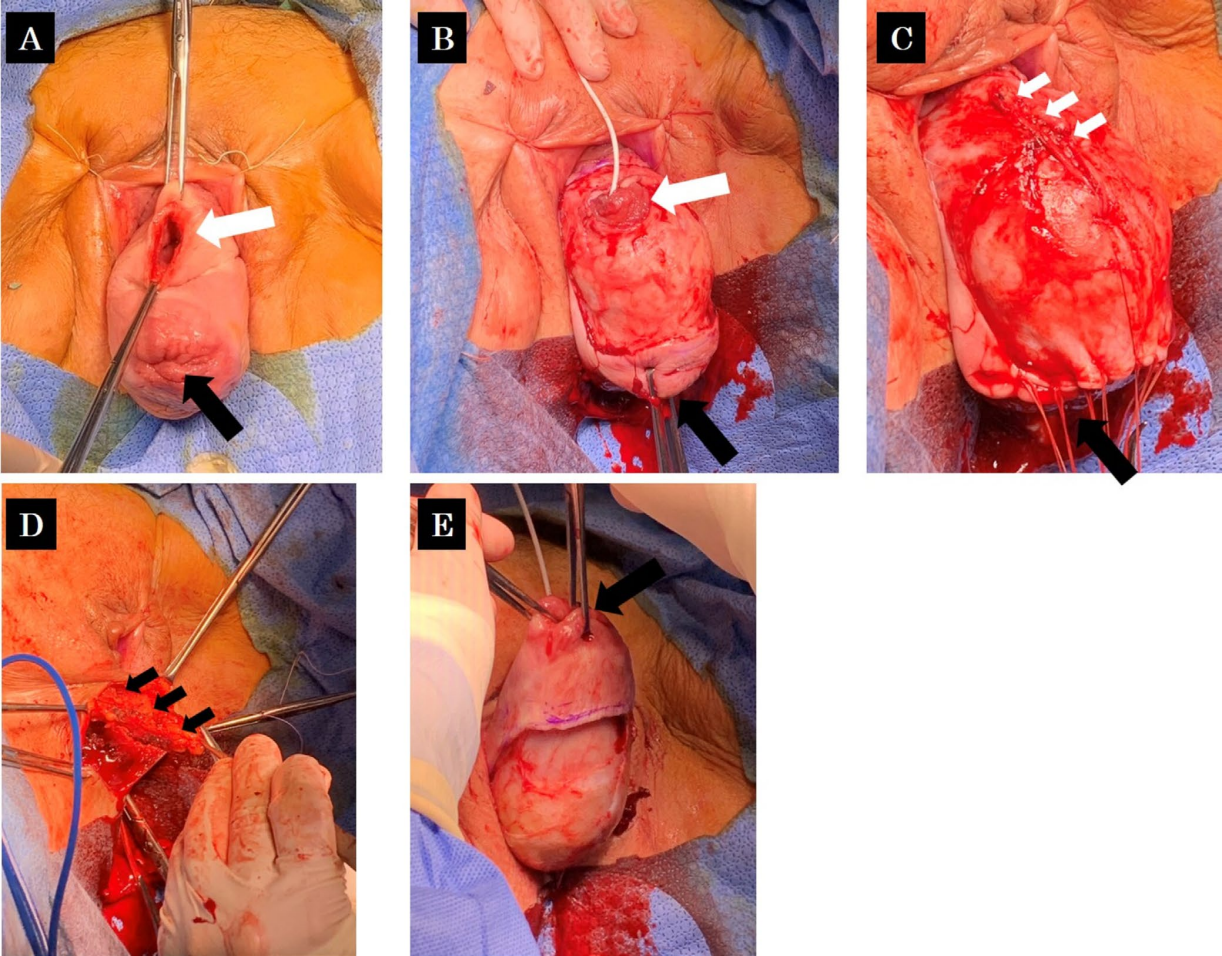
Surgical findings. (A) Complete uterine prolapse (black arrow) and vesicovaginal fistula (white arrow) after repairing everted bladder mucosa. (B) Removing of the anterior vaginal wall. The black and white arrows indicate the uterine and the fistula, respectively. The white catheter is inserted into the right ureter. (C) Closure of the vesicovaginal fistula in two layers (white arrows). (D) A labial fat pad (Martius flap) (black arrows) harvested from the patient's right labia. A vertical incision is made through the labia majora, and the subcutaneous tissue is dissected to mobilize the fat flap while sparing the vascular pedicle. The flap is positioned over the closure line. (E) Removing of the posterior vaginal wall. After that, the anterior and posterior vaginal walls were sutured together to close the vagina.

Therefore, we decided to perform a one‐stage surgical repair using a minimally invasive approach due to the patient's advanced age. We performed fistula closure and colpocleisis without the addition of a transvaginal hysterectomy. During surgery, a ureteral catheter was inserted into the ureter. After denuding the anterior and posterior vaginal walls, the VVF was closed in two layers (bladder mucosa and thin muscle, and the connective tissue between vagina and bladder). The closure line was reinforced with a labial fat pad (Martius flap) harvested from the patient's right labia majora. The anterior and posterior vaginal walls were then sutured together to close the vagina (Figure 3).

On the day after surgery, the patient developed myocardial infarction and underwent emergency percutaneous coronary angioplasty (PCA). Her post‐PCA course was uneventful. She was discharged on the 14th postoperative day. Five months after surgery, hydronephrosis had resolved, and her serum creatinine level had improved to 1.41 mg/dL. Now, 18 months postoperatively, she remains free of POP recurrence and urinary incontinence.

## 3 Discussion

Here, we present a case of bladder eversion through a VVF in a patient with complete uterine prolapse. The combination of POP, VVF, and bladder eversion, which primarily affects elderly women, can lead to urinary leakage, vulvar pain and bleeding, or renal dysfunction due to hydronephrosis, ultimately compromising the patient's quality of life. Closure of both the fistula and the vagina in a single operation is a minimally invasive and favorable surgical option for frail elderly patients.

The prevalence of POP varies depending on the population, geographic characteristics, and the definition or severity of POP. Studies have reported that the percentage of women with POP‐Q stage II or higher ranges from 11.8% to 60.9% [4, 5]. A recent Japanese study demonstrated that 28.7% of women aged 70 and older had POP‐Q stage II or higher [1]. In the United States, approximately 50% of women with symptomatic POP are aged 80 years or older [6]. The prevalence of POP is relatively high in this patient age group. Despite being aware of POP, she did not seek treatment for 2 years. This reflects a broader trend, as the number of patients who actually visit hospitals for POP is small. Luber et al. reported that the rate of women aged 70–79 who seek medical help for POP is only 18.6 per 1000 [7].

The most common cause of VVF in developing countries is necrosis due to prolonged obstructed labor, whereas in developed countries, it is typically associated with total hysterectomy, radiation therapy, pessary use, infection, and trauma. Although the exact cause of VVF in the present patient was unclear, local ischemia was suspected, given her advanced age, severe POP, and preexisting cardiovascular disease. Ting et al. also reported a similar case of severe POP complicated by a VVF with no other identifiable triggers [8]. Severe POP can lead to fistula formation due to ischemia of the vaginal mucosa, highlighting the need for timely intervention in such cases.

Transvaginal or transabdominal repair is a surgical option for both POP and VVF. Yang et al. presented a case of severe cystocele and VVF in which the patient underwent transabdominal VVF repair with omental interposition and the Burch procedure for cystocele correction [9]. While transvaginal and transabdominal VVF repairs offer comparable outcomes, the transvaginal approach has advantages such as minimal blood loss, shorter hospital stay, and relatively lower postoperative morbidity. Hadzi‐Djokic et al. reported 220 cases of VVF, in that they recommended the transvaginal approach for small and distally located fistulas, while the transabdominal approach is rarely required, and is used in complex cases, with great or recurrent fistulas, or in situations with an additional surgical procedure [10].

Martius flap was used in an effort not to recur the fistula in this case, but it is not clear that this was always necessary. The main indication for Martius flap are to reduce fistulae or recurrence in urethral diverticulectomy, urethral mesh excision or vaginal repair of fistulae, to prevent recurrent scarring of the urethra following urethrolysis or urethral stricture repair, or to protect the fragile urethra from stress incontinence surgery complications [11]. Alternative flaps such as peritoneum, gracilis, or omentum are slightly more invasive, however, Martius flap is very convenient because it can be used in the same transvaginal operative field [11].

There were few reports on bladder eversion through a VVF in a patient with complete uterine prolapse. The mechanism of bladder eversion is unclear. Bladder eversion through the external urethral orifice is thought to be caused by several factors, such as fragility of urethral tissues, fragile supporting tissues of the bladder, or chronic increase in abdominal pressure due to difficulty in micturition [12, 13, 14]. In the present case, bladder eversion occurred not through the urethral orifice; however, we speculate that the weakness of the bladder supporting tissues and increase in abdominal pressure for micturition could be the causes of bladder eversion through VVF. Similar cases reported previously have been treated without bladder fixation [9, 15, 16].

For this very elderly patient, we opted for a transvaginal procedure and covered the VVF closure line with a Martius flap. To minimize operative time and reduce blood loss, a hysterectomy was not performed; instead, colpocleisis was selected. Although the patient experienced a postoperative myocardial infarction, she was successfully treated. Therefore, less invasive surgical procedures with shorter operative times and less blood loss should be considered for severe POP and associated VVF, particularly in elderly patients, as these conditions may occur more frequently in this population.

## 4 Conclusions

We report a rare case of VVF with bladder eversion complicated by complete uterine prolapse, successfully treated with a one‐stage surgical procedure. In severe cases of POP, VVF may develop, highlighting the importance of early management to prevent such complications.

## Ethics Statement

The authors have nothing to report.

## Consent

Written informed consent was obtained from the patient.

## Conflicts of Interest

The authors declare no conflicts of interest.

## References

[1] J. Kato , C. Nagata , K. Miwa , N. Ito , and K. I. Morishige , “Pelvic Organ Prolapse and Japanese Lifestyle: Prevalence and Risk Factors in Japan,” International Urogynecology Journal 33 (2022): 47–51. 33580329 10.1007/s00192-021-04672-7

[2] P. Hilton and D. A. Cromwell , “The Risk of Vesicovaginal and Urethrovaginal Fistula After Hysterectomy Performed in the English National Health Service—A Retrospective Cohort Study Examining Patterns of Care Between 2000 and 2008,” BJOG: An International Journal of Obstetrics & Gynaecology 119 (2012): 1447–1454. 22901248 10.1111/j.1471-0528.2012.03474.x

[3] S. Rajaian , M. Pragatheeswarane , and A. Panda , “Vesicovaginal Fistula: Review and Recent Trends,” Indian Journal of Urology 35 (2019): 250–258. 31619862 10.4103/iju.IJU_147_19 PMC6792412

[4] J. T. Seo and J. M. Kim , “Pelvic Organ Support and Prevalence by Pelvic Organ Prolapse‐Quantification (POP‐Q) in Korean Women,” Journal of Urology 175 (2006): 1769–1772. 16600755 10.1016/S0022-5347(05)00993-6

[5] Y. S. Lien , G. D. Chen , and S. C. Ng , “Prevalence of and Risk Factors for Pelvic Organ Prolapse and Lower Urinary Tract Symptoms Among Women in Rural Nepal,” International Journal of Gynaecology and Obstetrics 119 (2012): 185–188. 22925819 10.1016/j.ijgo.2012.05.031

[6] J. M. Wu , C. P. Vaughan , P. S. Goode , et al., “Prevalence and Trends of Symptomatic Pelvic Floor Disorders in U.S. Women,” Obstetrics and Gynecology 123 (2014): 141–148. 24463674 10.1097/AOG.0000000000000057 PMC3970401

[7] K. M. Luber , S. Boero , and J. Y. Choe , “The Demographics of Pelvic Floor Disorders: Current Observations and Future Projections,” American Journal of Obstetrics and Gynecology 184 (2001): 1496–1501. 11408873 10.1067/mob.2001.114868

[8] N. S. Ting , H. C. Lee , J. Y. Ke , P. C. Li , and D. C. Ding , “Total Uterine Prolapse Complicated With Vesicovaginal Fistula: A Case Report,” Medicine 100 (2021): e26386. 34128901 10.1097/MD.0000000000026386 PMC8213252

[9] C. H. Yang , “Cystocele With Concomitant Vesicovaginal Fistula,” Incontinence and Pelvic Floor Dysfunction 4 (2010): 117.

[10] J. Hadzi‐Djokic , T. P. Pejcic , and M. Acimovic , “Vesico‐Vaginal Fistula: Report of 220 Cases,” International Urology and Nephrology 41 (2009): 299–302. 18810652 10.1007/s11255-008-9449-1

[11] A. Wilson , S. Pillay , and T. Greenwell , “How and Why to Take a Martius Labial Interposition Flap in Female Urology,” Translational Andrology and Urology 6 (2017): S81–S87. 28791226 10.21037/tau.2017.04.38 PMC5522801

[12] J. H. Kim , D. Y. Cho , J. H. Bae , and H. S. Park , “Complete Bladder Eversion Concurrent With Total Uterine Prolapse,” International Urogynecology Journal 21 (2010): 503–505. 19936594 10.1007/s00192-009-1017-4

[13] C. M. Kalorin , J. Belarmino , B. Mian , and E. De , “Complete Eversion of the Urinary Bladder: Presentation, Review, and Algorithm for Management,” Obstetrics and Gynecology 113 (2009): 496–501. 19155933 10.1097/AOG.0b013e318184ee8b

[14] M. A. Mastropietro , M. H. Clark , and D. S. Hale , “Transurethral Bladder Eversion Concurrent With Uterovaginal Prolapse,” Obstetrics and Gynecology 99 (2002): 921–925. 11975960 10.1016/s0029-7844(01)01669-6

[15] N. Fennimore , S. H. Boyles , and M. A. Denman , “Bladder Eversion Through a Vesicovaginal Fistula in a Patient With Uterine Procidentia: A Case Report,” Female Pelvic Medicine & Reconstructive Surgery 28 (2022): e231–e232. 35421010 10.1097/SPV.0000000000001176

[16] S. Basak and T. S. Bag , “Corrosive‐Induced Vesicovaginal Fistula With Procidentia: A Rare Occurrence,” International Urogynecology Journal and Pelvic Floor Dysfunction 19 (2008): 1719–1721. 18521527 10.1007/s00192-008-0652-5

